# Legal Notes for Institutional Workers

**Published:** 1911-12-30

**Authors:** 


### LEGAL NOTES FOR INSTITUTIONAL WORKERS.

(The Editor will be pleased to answer Legal queries in this column.)

### THE INSURANCE BILL AND THE VOLUNTARY HOSPITALS.

The Insurance Bill having been passed by the House of Lords substantially in the form in which it left the House of Commons, it is time to consider whether anything took place during the last phases to abate its rigour as against the voluntary hospitals. Alas! There is nothing to record. The clauses to which attention was drawn in these notes a few weeks since have not been modified, and they are now sections of an Act of Parliament. Substantially, the voluntary hospital is without the pale of the scheme; and those who are responsible for institutional management must wait—like Micawber—for something to turn up to relieve their anxieties. It seems that they may have to wait until those who now frequent the out-patient department, or clamour for accommodation in the wards begin to find that the wells of charity have been dried up.

### THE POWERS OF THE DOCTORS.

A careful perusal of those portions of the Act which relate to medical benefit is sufficient to show that a united medical profession has power to modify the operation of the scheme in one very important respect. Assume, for instance, that the doctors in the district of an Insurance Committee objected to medical benefit being given to persons whose income exceeded £100, and refused to allow their names to be placed upon the panel of doctors. If the Committee had prescribed no income limit, it would then be left to the Insurance Commissioners to provide for medical benefit in some other way; but the funds at their disposal could not exceed the amount which would have been expended on medical benefit. On the other hand, if the Insurance Committee were to fix an income limit of—say £130—which was not acceptable to the doctors, the Insurance Committee would be entitled under Clause 15 to require all persons having incomes over that amount to make their own arrangements for medical benefit, and to allow all other persons to do so. To enable him to make his own arrangements, the amount which would have otherwise been expended on medical benefit would apparently be handed to the insured person direct.

### EFFICACY OF A STRIKE OF THE MEDICAL PROFESSION.

The doctors, then, can effect a reversal of the policy of the Act. They can prevent medical benefit being conferred through practitioners employed and paid by the State. It is clear, however, that there might have to be unanimous agreement on the part of the medical profession. Mere agreement amongst the practitioners in a particular district would not necessarily be effective, because the "panel" for a district need not be chosen solely from the doctors practising in that district. Of course, a doctor in Newcastle-on-Tyne would not be likely to place his name on the panel for the City of Westminster; but it is possible that a sufficiently good panel might be obtained from amongst the doctors on or near the border-line between two districts.

### WOULD THE POSITION OF THE HOSPITALS BE AFFECTED?

Stating the result of what has been written above in a sentence, it seems that the doctors practically have it in their power to compel the State to administer medical benefit by a simple increase of the weekly payment to insured persons who are on the sick list. Assume that this change could be brought about, would the hospitals benefit in any degree? It would seem to leave them in the same hopeless position as before. There is not much reason to think that an insured person receiving, say, 12s. 6d. a week would contribute anything more to the hospitals than a person who receives 10s., seeing that persons of this class never have been used to contribute anything at all. In the meantime the sources of supply continue to be drained by the taxes imposed upon those who, at the present time, are wont to contribute something to charity as administered in the great hospitals of the country.

### COMMUNICATIONS BETWEEN DOCTOR AND PATIENT.

The medical practitioner is probably familiar with the rule of law under which, notwithstanding the oath of Hippocrates, he may be compelled to reveal in the witness-box the confidences of the consulting-room. He may complain, with some show of reason, that whereas the lawyer need not disclose what passed between him and his client, communications between doctor and patient are in no sense privileged. The reason is that as legal assistance is sanctioned by law, no litigant would be safe if he or his lawyer were compelled to state what passed in consultation. There is a tendency, however, to relieve the doctor from making any revelation with regard to his patient unless it is absolutely necessary; and one County Court judge, in the course of a recent case, for this reason deprecated a question which was asked of a medical practitioner. Perhaps the Legislature may one day intervene in the matter. It is well for the practitioner to remember that while he may be compelled to state what his patient tells him, all that is told may not be evidence. For instance, in a recent compensation case a doctor was asked to describe the symptoms of the claimant. He explained the symptoms, and was then asked: "What was the cause?" "The claimant told me," he began. This question was disallowed, on the ground that it was mere hearsay. Doubtless he left the court wondering at this curious intervention; but the reason why hearsay is inadmissible is that the witness is stating something which was not said on oath and upon which there can be no cross-examination.

